# The State of Lunacy in Ireland

**Published:** 1863-10

**Authors:** 


### ART. IV.—THE STATE OF LUNACY IN IRELAND.

THE Twelfth Report on the District, Criminal, and Private Lunatic Asylums in Ireland, which has just been published, presents us, in a very clear and comprehensive style, with a view of the condition of a large portion of the insane population of the sister country, and offers, at the same time, many valuable suggestions as to modes in which that condition may be improved. It is, in fact, a very interesting and practical Blue Book, unmistakeably showing that the Irish Inspectors, by whom it is compiled, and of whose work it is a record, discharge the important duties committed to them in the most painstaking and temperate, yet earnest manner. But still this Irish Report is, in our eyes, an imperfect production, and will continue to be so until it gives us an account of those lunatics, known as "*single cases*," residing with relatives, friends, or strangers, equally minute with that which it now furnishes regarding their fellow sufferers in asylums, poor-houses, and gaols. Those unconfined insane in Ireland number no fewer than 8384 persons, and thus considerably exceed the confined insane, who are only returned as 7862; while all the information we can gather regarding them is of the most meagre and unsatisfactory description. The returns regarding them, derived entirely from the constabulary of the various counties and the Dublin metropolitan police, are doubtless accurate so far as they go; but they refer solely to number and distribution, and are silent as to what we would most desire to know about, namely the circumstances in which these unfortunate lunatics are placed. We are fully persuaded that it is the unconfined insane who require supervision and protection by competent authorities far more than their incarcerated brethren. They are peculiarly exposed, by their very position, to neglect and cruelty, which may be practised against them with little fear of detection; and we cannot, therefore, regard any lunacy system as perfect or complete which does not provide for their regular inspection, and for periodic reports upon them, similar to that which is now before us with reference to those lunatics shut up in asylums. A general view, too, of lunacy in Ireland as a whole, would of course be of far greater value than the partial though excellent survey which is now provided.

The Report of the Inspectors commences with a general summary, in the form of a tabular statement, of the whole insane population of Ireland, and how they are at present located, contrasting the past with the previous year, excepting as regards the lunatics at large, respecting whom information was not obtained in 1861. From this table we learn, that on the 31st of December last there were in Ireland 16,246 lunatics—8541 males and 7705 females, of whom, as we have said, 8384 were at large and 7862 in confinement. The total of the insane population cannot be compared with any figures representing it in the previous year, owing to the want of returns as to unconfined lunatics; but the total number of confined lunatics is found, on contrast, to be slightly less than that for 1861, being 7862 in the one case against 8055 in the other. The diminution has taken place among the insane inhabitants of poor-houses and prisons, for the population of asylums, public and private, has undergone an increase. The returns of the district asylums exhibit an increase of 118—103 males and 15 females. The distribution of the confined insane is given as follows:—In district asylums, 4506; in poor-houses, 2225; in gaols, 378; in private asylums, 546; in the central asylum, 128; in Lucan Spa Asylum, 77; and in convict prisons, 2.

After this preliminary statement the Inspectors go on at once to describe individually the various district asylums, carefully noting their actual condition, any changes in their constitution or management which may have been effected in the past year, with the general results of the treatment adopted in each. The accounts given of these asylums ought to be trustworthy in no ordinary degree, as they are not founded upon any single visit, but upon a series of inspections, all of the asylums having been subjected to supervision at least five, and some as many as eight times in the course of a twelvemonth. It is gratifying to know that the large majority of the asylums receive the cordial approval of the Inspectors, while all of them afford some ground for commendation. From each report we could extract, did space permit, some pleasing and instructive passage, all indicating that the Irish asylums are rapidly advancing to a high state of efficiency; and that in many of them efforts are at present being made to minister to the comfort of their inmates, by alterations in their structure, and by the introduction of modern improvements in their furnishing and arrangements. Thus, in their notes on the Armagh Asylum, the first in the list, we find the Inspectors saying:—

"The changes referred to in the last Report as being contemplated for the improvement of this asylum, have made rapid progress. The additions and alterations were commenced in 1862, and though still in the hands of the contractors, are in a very advanced state—so much so, that the large dining-hall, sixty feet by thirty, has been already availed of for purposes of recreation. The new laundry and kitchen—departments on the suitability of which much depends—are nearly completed, as are also the water-closets, baths, and lavatories, and an engine for pumping water, which will further supply steam for cooking purposes."

In the Report on the Limerick Asylum it is observed :—

"The new chapel for the use of the patients was completed in all but the furniture and fittings, and the inspectors accordingly made suggestions with a view to having suitable arrangements made for the celebration of divine service. A Turkish bath, which is just about to be opened, is also among the improvements at this asylum."

At the Richmond Asylum for the County and City of Dublin and Counties of Louth and Wicklow—

"Various improvements, structural and other, have been effected in the course of the year, amongst which may be enumerated a new cooking apparatus and lavatories. The drying-house, which had been burned down, was also rebuilt, and the whole house underwent a general painting."

Regarding the Sligo Institution we read :—

"We expressed in our minute our gratification at the erection of the fine refectory recently completed, and which has also been occasionally used as a recreation-hall."

In several asylums, as for example Belfast and Richmond, overcrowding and want of sufficiently ample accommodation are complained of; but this is likely to be remedied soon, by the opening of the new asylums which are being built, and the additions which are being made to existing institutions, in which provision is afforded for 2000 lunatics.

Throughout their Report the Inspectors take every opportunity of urging, what has been so often and earnestly recommended in these pages—the early removal of lunatics to asylums where they can be placed under judicious treatment at the very time when the malady under which they labour is most capable of cure. The statistics of the Cork Asylum, over which Dr. Power so well presides, illustrate this in a marked way. There the Inspectors found that of 94 persons discharged recovered, 62 had been less than a month ill previous to admission, 16 under three months, and 10 less than a year. In the table, too, attached to the Report we observe that, of all the cases of recovery discharged during the year, 554,424 individuals had been less than twelve months ill. In their summing up on this subject the Inspectors say :—

"It cannot therefore be too often repeated, nor can the knowledge be too widely diffused, that on the first manifestations of derangement, the prompt removal of the patient to an asylum affords an almost certain hope of speedy recovery, while the opposite course will as assuredly lead to confirmed and incurable insanity."

To this they add :—

"There is another point deserving of notice, in reference to recoveries, and that is, the large number discharged after a short term of treatment—410 out of the 554 cases having recovered within periods varying from one to twelve months."

The statistics throughout the Report are elaborate and instructive, and deal with a great variety of topics. Let us take one or two examples from the Report on the Belfast Asylum, under the able superintendence of Dr. Stewart :—

"There were 357 patients resident on the 31st December, 1861—193 males and 164 females; 92 admissions occurred during 1862, making a total of 449 under treatment for the year—236 males and 213 females; the discharges and deaths amounted to 84, leaving 365 resident at the end of the year, being 8 more than at the corresponding period of the preceding year. There were 57 patients discharged cured and 9 improved, the cures being 61·9 per cent. on the admissions, which is an exceedingly high average. Eight of the admissions were by the authority of the Lord Lieutenant, being transferences from gaols in which the patients had been confined as dangerous lunatics, but as regards whom the medical superintendent reports that they did not differ much, if at all, in their symptoms, &c., from the ordinary class of admissions. Eighty-four persons were admitted on the authority of the Board."

"The ages of 43, or nearly half the number admitted, ranged from under twenty to thirty years; 44 from over thirty to sixty; and 5 from sixty to seventy. Of the admissions, 69 were cases of mania, 12 monomania, 9 melancholia, and 2 dementia. 233 cases of mania remained in the asylum at the end of the year; 24 of mania complicated with epilepsy, 32 monomania, 67 melancholia, and 9 dementia. Only 13 of the whole number resident are returned as well educated; 26 can read and write well; indifferently, 139; and 116 who can read only; the number who cannot read or write being 71. The education as regards the sexes appears nearly equal—only 40 males out of 200 being illiterate, and 31 females out of 165."

"The single predominate largely, and the proportion of the sexes is pretty equal. The numbers of the married also bear a not unequal proportion—viz., 60 males to 43 females; the widowers amounting to 11, and the widows to 13. The classification of the inmates as regards their conduct and demeanour is as follows:—Convalescent, 78; quiet and orderly, 103; moderately tranquil, 113; noisy and refractory, 71. Thus the number of noisy and refractory patients bears but a comparatively small proportion to the three other denominations. The imbecile and epileptic amounted to 60, and there were 24 patients with suicidal tendencies. The state as to probability of recovery of those resident is set down as follows: probably curable, 99; probably incurable, 235; epileptics, 31."

These examples, extracted from a large mass of numerical facts having reference to the Belfast Asylum, show how copious are the statistics contained in the Report. In the quotations which we have selected, there may be traced the careful hand of Dr. Stewart, to whom, indeed, the Inspectors acknowledge their obligations. But in addition to the special statistics given under the head of each asylum, there are also appended to the volume a large number of statistical tables, conveying much information upon a variety of topics. From these may be learned the number of lunatics, idiots, and epileptic imbeciles unconfined, in the different counties and constabulary districts in Ireland; the number of insane of all classes who have received relief in workhouses during the past year, with the number who have died or been discharged; and the aggregate of inmates, the admissions, discharges, and deaths in the district asylums during the same period. We have also tables referring to the ages of patients admitted into asylums, the duration of disease previous to admission, the length of the term of confinement, the number of relapses, and a great many other interesting questions. In that regarding the causes of death we find that 361 lunatics died in the public asylums of Ireland in 1862, and that of these, 52 died of abdominal affections; 113 of cerebral affections; 106 of diseases of the lungs; 10 of diseases of the heart; 59 of debility and old age; 13 of other diseases; and 8 from violence, accident, or suicide. In discussing the last-mentioned causes of death, or the casualties of the year, the Inspectors express a regret that they have been more than usually numerous, but also entertain a hope that the precautionary measures which have been adopted "and particularly the introduction of regular night nursing," will have the effect of providing, as far as human foresight will permit, against the recurrence of such untoward events. It would thus appear that "night-nursing" has not been hitherto regularly or systematically carried out in all the Irish asylums. To what extent it has been practised we are unable to ascertain, but we earnestly trust that in future it will be universally adopted, and that the Inspectors will see that all the institutions under their care are provided with night-watchers —officials whose usefulness it is impossible to over-estimate, who are valuable in bringing quiet, and confidence, and slumber to many an anxious and timid patient; in visiting the sick and administering medicines; in promoting cleanliness and comfort; in carrying out those observations which are guides to treatment, and in preventing catastrophes such as have had to be deplored in two Irish asylums in the past year.

The table as to the form of disease in those lunatics in district asylums on 31st of December last shows that of the total number, 4506, 2511 suffered from mania, 647 from melancholia, 626 from dementia, 231 from monomania, 61 from imbecility, 146 from idiotcy, and 284 from mental affections, complicated with epilepsy. Here we are at once struck with the great disproportion of cases of mania, which amount to more than one half of the whole number under treatment. Perhaps national character has something to do with determining the form of mental disease to which a people shall be chiefly liable. It is clear that the type of insanity has not changed in Ireland, as some think it has in England, of late years, and this probably, together with the violent character of the mania which has to be treated in Ireland, is the cause why the camisole is more frequently employed there than here.

The table exhibiting the cause of the mental derangement in the patients under treatment, traces it to poverty and reverse of fortune in 206 instances; to grief, fear, and anxiety, in 264; to love, jealousy, and seduction, in 118; to domestic quarrels and afflictions, in 54; to religious excitement, in 124; to study and mental excitement, in 50; to ill-treatment, in 15; to pride, in 6; to anger, in 8; intemperance and irregularity of life, in 271; to cerebral disease, in 253; to congenital defects, in 58; to febrile affections, in 39; to effects of climate, in 61; to bodily injuries and disorders, in 182; to abuse of medicine, in 13; and to hereditary predisposition, in 505. The cause was unknown in 2279 cases.

From the table recording the previous occupation of the asylum inmates, we learn that the largest proportion, 1284, belonged to the labouring classes; while 637 were domestic servants; 372 had been engaged in farming; 180 as tailors and seamstresses; 70 as shopkeepers; 96 as soldiers; 64 as artisans; 64 as pedlars; 46 as mendicants, &c. &c. Of the professions we find there had been in confinement, in district asylums, 4 lawyers, 5 medical men, 58 students and teachers, and 25 members of religious communities. To the enormous number of 1144, no occupation is assigned.

Of the inmates of the district asylums, 992 were married; 3089 single; 258 widowed; while the social condition of 167 was unknown. Relationship between inmates was traced only in 172 instances, and in these every degree of relationship was embraced; 1135 patients are returned as probably curable, and 3371 as probably incurable.

The next set of tables to that which we have just been examining refers to expenditure. Upon this subject the Inspectors say:—"The expenditure of the various asylums has, on the whole, been regulated with due regard to the comforts and necessities of the insane." The lowest annual charge is at Cork, where 16*l.* 18*s.* 6*d.* is the average of expenditure per patient; the next lowest at Omagh, where it is 17*l.* 19*s.* 11*d.*; and the highest at the Metropolitan Asylum at Richmond, where it amounts to 26*l.* 9*s.* 2*d.*, which is more, however, than the annual charge at Clonmel only in the sum of 2*s.* 8*d.* The mean average cost of each patient per head per annum, on the entire outlay for the year in Ireland, is 21*l.* 2*s.* 8*d.* The salaries of the medical officers, which are also tabulated, seem, in some instances, unnecessarily small, and the wages allowed to nurses and attendants must appear exceedingly low to those accustomed to the English tariff. The attendants receive from 21*l.* to 8*l.* per annum; 12*l.* or 14*l.*, with rations and clothing, being considered a fair remuneration. The nurses receive from 10*l.* to 5*l.* per annum. While speaking of these officials, we may mention that we have noticed with regret that the old term, "Keeper," of zoological suggestion, is still in use in some parts of Ireland, and that in one asylum, the scarcely less objectionable title of "Warder" has been introduced. It is to be regretted that we have not an appellation for those officers in charge of male patients equally good with the word "Nurse," applied to their colleagues in the female department. Attendant seems to be the best term at present at command, and is certainly superior to keeper or warder. This may, at first sight, appear a matter of great insignificance, but it is, in reality, of some importance, for many of the insane are morbidly sensitive, and are keenly alive to iron bars, jingling keys, and harsh-sounding words, which savour more of a prison or an ancient madhouse than of a modern hospital for the insane.

Leaving the mass of statistical matter which the Inspectors have collected, and upon which we have only briefly touched, and reverting to the body of the Report, we come upon a short, but on the whole satisfactory, notice of the Irish Licensed Houses, and a lengthened description of the condition of the Central Criminal Asylum at Dundrum. Of the cleanliness, order, and good management of this establishment the Inspectors speak highly. They observe—

"The principal event of the year was the removal of seventeen patients to district asylums. These persons had been tried and found guilty of various offences, and sentences of imprisonment or penal servitude passed upon them, subsequent to which, having become insane, they were transferred to the Central Asylum, under the 12th section of the Act 8 and 9 Vict. c. 107. On the expiration of their sentences, if sane, they would have been entitled to their discharge; but continuing to be of unsound mind, it was obvious that they could not be set at liberty without endangering their own safety or that of other persons; and having undergone the full term of their sentences, they ceased properly to be chargeable to the funds of a Government institution, or to be fit subjects for detention therein. The only course that remained, therefore, was to make arrangements for their transference to the district asylums to which, as pauper lunatics, they appeared to have been admissible before they were taken into custody, particularly as a number of lunatics eligible for admission into the Central Asylum were detained in several of the gaols, and in the convict prisons, awaiting vacancies."

The Central Asylum is about to have a considerable addition built to it, affording accommodation for 50 patients. A Turkish bath is also to be added.

In commenting on the dietary of the Irish asylums, the Inspectors have in several instances to complain as to its insufficiency, or to give but a qualified approval of it. In many of the institutions, however, it is on a liberal scale. Taking Limerick as an example of one of the best dietaries, we find that the patients there are allowed meat five times in the week; 12 oz. to males on Sundays and Thursdays, with a pint of soup and 4 lbs. of potatoes or 8 oz. of bread; on the other three days, 8 oz. of meat for males and females, with a pint of soup and 12 oz. of bread for males and 8 oz. for females; with bread and milk in sufficient quantity on the two remaining days, —viz., Wednesdays and Fridays. The dietary in that asylum on 31st December last was as follows:—

"*Ordinary.*—Breakfast, 7 oz. of Indian meal made into stirabout, or 12 oz. of bread for the male, and 8 oz. of bread for female patients, new milk and tea. Dinners: 12 oz. of beef, and 12 oz. of bread or 3 lbs. of potatoes, for males; and 2½ lbs. of potatoes or 8 oz. of bread, for females; milk. Supper: 6 oz. of bread and ½ pint of new milk, for male and female patients.

"*Extra.*—Breakfast: tea, butter. Dinner: roast beef, mutton.

"*Hospital Diet.*—Wine, porter, spirits, eggs, butter, tea."

When referring to amusements, the Inspectors remark:—

"In the great majority of the asylums the amusements and occupations of the patients are now objects of much interest with the officers placed in charge of them; indeed, with two or three exceptions, which we have referred to more particularly in the course of this Report, the Irish Asylums are, on the whole, very well provided for in this respect; and at the Metropolitan, Belfast, Sligo, Kilkenny, and some others, no pains have been spared in ministering to the enjoyment of the inmates."

Believing, as we do, that those amusements which engage the attention and awaken interest, without producing excitement, are among the most important of the agents by which we may operate directly upon the mind itself, and ought to constitute a leading feature in all efforts at treatment, we rejoice to learn that they are now so efficiently conducted in the Irish asylums; and we rejoice the more, because there is reason to believe that, until very recently, they had not in these establishments received that consideration which they undoubtedly merit. An admirable pamphlet,* from the pen of John A. Blake, Esq., Member for Waterford, has been lately devoted to a discussion of this topic, and is so instructive in many respects, that we would recommend a careful perusal of it to all interested in lunacy affairs. Mr. Blake is a legislator who, unlike many with whom he is associated, does not "narrow his mind" and give up to party politics those powers which were meant to be employed for the moral and intellectual advancement of his species; but who, on the contrary, applies himself, with no common vigour and intelligence, to the good work of social reform. The pamphlet at present before us affords abundant proof of this, and exhibits, in a striking manner, the industry, acuteness, and wide and kindly sympathies of which it is the offspring. It is professedly an exposition of the "defects in the moral treatment of insanity in the public lunatic asylums of Ireland," and commences by sketching, in a brief but graphic manner, the dark ages of asylum government, and the negligences and atrocities which preceded the advent of Pinel, traces of which are still to be occasionally encountered. It proceeds to describe the rise and progress of the humane system, and the gradual substitution of moral for the worst kind of physical treatment; and then, having presented a series of quotations from the writings of the highest medico-psychological authorities as to the efficacy of amusement and occupation in the treatment of mental disease, it leads evidence as to the condition of the Irish asylums with respect to these in 1856, at the period of the Royal Commission. This evidence, which consists altogether of the well-weighed statements of medical officers of asylums, certainly justifies Mr. Blake in declaring "that a very great deficiency existed at the period of the Commission in the way of either system or appliances for occupying and amusing the patients." So strong is the evidence, that we think it renders quite unnecessary the explanation given by Mr. Blake as to the contrast which he is said to have instituted between the English and Irish asylums; for had he declared, in the most emphatic terms, that "the English asylums, taken as a whole, were better than those in Ireland" in so far as amusement and occu-

---

* *Defects in the Moral Treatment of Insanity in the Public Lunatic Asylums of Ireland, with Suggestions for their Remedy, and some Observations on the English Asylums.* By John A. Blake, M.P. London: 1862. pp. 103.

pation are concerned, he would have been unquestionably in the right. As illustrative of the state of the Irish institutions in these respects, we give one or two extracts from the Report of the Royal Commissioners. In the evidence of Richard Tuohill, M.D., of the Richmond Asylum, we find—

"Are any arrangements made for providing amusement for the patients? Very little; not sufficient. I have given a report suggesting the propriety, in the system of moral treatment, of having intellectual and pleasurable amusements. That has not been carried out."

In that of Mathew E. White, M.D., of the Carlow Asylum—

"Are any amusements provided for the patients? They read and walk.—Is there a library of books for their use? Well, there was, but it is gone: the books are torn. It is twelve or fifteen years ago since there was about 5*l.* worth of books bought, and they are not now forthcoming."

And in that of J. B. M. Kiernan, Esq., manager of the Ballinasloe Asylum—

"What means of amusement are there on wet days for the large number of inmates in this house? Nothing particular.—Are there any prints or anything on the walls to relieve the monotony of the place? No."

We venture to assert that these statements which we have quoted, would not have been found applicable to any English or Scotch public asylum in 1856; and here indeed we feel called upon to remark that, as regards the latter (the Scotch asylums), Mr. Blake has fallen into grave error. When speaking of the system of moral treatment followed in the Irish asylums, he says—"True, it may be better than that pursued in some other places (certainly far beyond that of Scotland a few years ago, where in many instances the barbarous cruelty practised towards lunatics would have disgraced the fourteenth century)," &c.

Now the real fact is that the investigations of the Royal Commissioners in 1855 showed that the public or chartered asylums of Scotland were in a very creditable and satisfactory condition, no suspicion of cruelty being alleged against them; and proved at the same time that they had taken the lead in all forms of amusement as a branch of treatment. Some of them indeed, as for example the Crichton Royal Institution, Perth, and Montrose, have always been distinguished for the immense number and variety of entertainments and employments which they provide for their inmates; and we have often thought it remarkable that it was on the north of the Tweed that private theatricals, costume balls, and Christmas-trees were first introduced into asylums. It was in the private asylums of Scotland, and in the houses where single cases were detained, that cruelty was detected, and not in the noble charities which have been founded in that country for the benefit of the insane.

From the time at which the evidence which we have adduced from the Report of the Royal Commissioners in Lunacy for Ireland was given, great strides have certainly been taken in the right direction by the Irish asylums, and much has been done to supply the wants which were then indicated. The Report of the Inspectors for last year informs us that in many of the asylums there are large numbers of books, periodicals, and newspapers in circulation, and that there are besides all sorts of indoor amusements, such as bagatelle, backgammon, cards, chess, draughts, dancing and singing; with out-door games, such as croquet, foot-ball, hand-ball, aunt Sally, nine-pins, &c. But much still remains to be done, much must be done, before all the Irish asylums are brought up to that standard which Mr. Blake very properly sets up; and much may be done we confidently predict, without incurring that risk which has filled with apprehension a wiseacre quoted by Mr. Blake, who feared to increase the enjoyments of the patients in his asylum, lest the sane people outside should feign madness in order to be admitted to participate in them. There can be no doubt, however, that the Irish asylums have been and are steadily progressive. Any defects which may have characterized them have not been attributable to the Inspectors, who obviously entertain the most enlightened and liberal views; not to the medical officers, for the Irish superintendents, from all we can learn of them, are a body of energetic and philanthropic men; but to the local boards, whose parsimony and prejudice have defeated many a benevolent effort. And it is therefore that we hail Mr. Blake's pamphlet with peculiar pleasure. Himself a member of a managing board, free from professional bias, and occupying a distinguished position, he speaks with no ordinary weight; and he has spoken so well as to enforce attention. In bringing his pamphlet to a conclusion Mr. Blake says—

"I trust the Chief Secretary will be induced to make further efforts for a class for whom he has shown an interest. The local governing boards can also do much to assist, by exercising the functions entrusted to them, and seeing, in addition, to the asylum being clean and orderly, the accounts regular, and the contracts fulfilled, that the few simple principles which I have already alluded to with regard to the moral treatment are in operation. It will not be out of place to remark, whilst on the subject, that committees would be much increased in efficiency by Government substituting other members for those who rarely attend, and establishing some rules by which an absence from a certain number of meetings during the year, without satisfactory reason being given, should, as a matter of course, disqualify from being longer a member of the Board. If the Inspectors would, in addition to their useful table of members who attend meetings during the year, also publish one containing the names of those not attending, I think it would be seen that a very large number habitually neglect their duties. . . . .

"I desire that it should be particularly understood that I have not ventured lightly to occupy public attention with this subject, without first endeavouring to make myself thoroughly acquainted with it. Apart from other considerations, I felt bound to do this to enable me properly to discharge my duties as one of a local board of management of an asylum, as well as a member of the legislature, where so many questions affecting the insane are discussed. During the last session, three important Bills relative to them have been passed. In addition to having visited nearly every asylum in the kingdom, I have gone over some of the principal ones on the Continent, consulted the best works, and have had the advantage of personally conferring with many of the men who have proved most successful in the treatment of insanity. My object is to make the public mind comprehend and adopt the great truths laid down on the subject, and especially to impress these truths on the minds and consciences of those who are placed in a position to alleviate the condition of those whom Providence has visited with the sad consequences of insanity. For until then we cannot hope to see accomplished for those afflicted ones all that skill judiciously directed can effect.

"Should I ever again allude to the subject, I have a cheerful hope that it will be to state that those responsible for the treatment of the insane in Ireland are discharging their high mission as they ought—for there cannot be a loftier aim than to be instrumental in alleviating the sorrows of a mind diseased, and aiding to bring it back to health and peace and usefulness. I hope I can then record that I found the patient surrounded by pleasing objects, calculated to withdraw him from the contemplation of his lingering delusions, and that no pains were spared to make him 'feel like a man amongst men,' and that nothing was neglected that could promote healthy thought and action."

These are sound views which cannot be too strongly insisted on, or too often repeated. Cordially do we trust that they may be generally adopted by the members of the visiting committees of the Irish asylums, and that the anticipations which Mr. Blake holds out may be more than fulfilled; so that very shortly there may be no Irish asylum second in means of moral treatment to Bethlehem, Derby, or Leicester, which Mr. Blake has placed before them in the meantime as models.

